# Effect of gut microbiota in the colorectal cancer and potential target therapy

**DOI:** 10.1007/s12672-022-00517-x

**Published:** 2022-06-24

**Authors:** Junchuan Li, Yuzhou Zhu, Lie Yang, Ziqiang Wang

**Affiliations:** Gastrointestinal Center, West China Hospital, Sichuan University, Chengdu, Sichuan China

**Keywords:** Gut microbiota, Colorectal cancer, Gut dysbiosis, Methylation, Tumor proliferation, Pathogenesis

## Abstract

The symbiotic interaction between gut microbiota and the digestive tract is an important factor in maintaining the intestinal environment balance. Colorectal cancer (CRC) is a complex disease involving the interaction between tumour cells and a large number of microorganisms. The microbiota is involved in the occurrence, development and prognosis of colorectal cancer. Several microbiota species have been studied, such as *Fusobacterium nucleatum* (*F.* *nucleatum*), Enterotoxigenic *Bacteroides* *fragilis* (ETBF), *Streptococcus bovi*s (*S. bovi*s), *Lactobacillus*, and *Bifidobacterium*. Studies about the interaction between microbiota and CRC were retrieved from Embase, PubMed, Ovid and Web of Science up to 21 Oct 2021. This review expounded on the effect of microbiota on CRC, especially the dysregulation of bacteria and carcinogenicity. The methods of gut microbiota modifications representing novel prognostic markers and innovative therapeutic strategies were also described.

## Introduction

CRC ranks third in the incidence rate and second in the mortality rate among all types of malignant tumours worldwide [1]. CRC is a heterogeneous cancer arising from different genetic and epigenetic events. Generally, CRC is considered a multifactorial disease caused by a high-fat diet, obesity, smoking, drinking, inflammatory processes, environmental factors and genetic alterations. CRC has a trend of increasing morbidity and mortality in developing countries. Recently, several studies revealed that CRC is significantly associated with microbiota features and diet [2–9]. The mode of life and diet seem to be the most important natural factors impacting the gut microbiota. Diet varies among ethnicities, nationalities and regions (rural or urban) [10]. The morbidity of CRC in different populations worldwide may be related to gut microbiota disorders in different regions. Evidence shows that the gut microbiota has a causal relationship with colorectal carcinogenesis [11]. Gut barrier dysfunction and increased tight junction permeability precede the development of colon tumors [12].

## General overview of the gut microbiota

The human gut microbiota is a complex community of bacteria, archaea, viruses and eukaryotes that is subject-specific and stable in healthy people [13]. Patients with CRC harbour a distinct microbiota. Scanlan et al. analyzed the fecal flora with CRC and colorectal polyps separately and observed an increased number of *Clostridium globosa* and *Clostridium tenderis* [14]. Dejea et al. observed that precancerous polyps from patients with familial adenomatous polyposis possessed biofilms with *E.* *coli* and *B.* *fragilis*, which can have a role in tumour progression [15]. These pathogenic bacteria were increased in patients and contributed to CRC by inducing tumour proliferation, promoting inflammation and DNA damage and protecting cancer from immune attack [16, 17]. Dai and Yachida found that probiotics, including *Bifidobacterium* and *Lactobacillus*, decreased, which exerted a protective effect against CRC [14, 16, 18]. With the advent of DNA sequencing technology, the capability to study microbiota composition has increased, allowing for more accurate and rapid identification and classification of microbiota in individuals. Metagenomic sequencing for large-scale fecal samples and mucosa indicated that gut microbiota diversity in CRC patients decreased [19, 20]. This phenomenon was also found in the case of intestinal adenoma [21]. Therefore, the relative abundance and proportion of gut microbiota are crucial for maintaining the intestinal microecosystem, which is a system with complex interactions among gut microbiota species, metabolites and intestinal tissues. The microbiome imbalance is associated with various diseases, including obesity, Crohn’s disease and gastrointestinal malignancies. A growing amount of evidence has supported that the environment and microbiome are risk factors for several malignancies, including CRC [22, 23]. Pathogenic bacteria can also cause tumours under specific conditions in mouse studies [24, 25]. Other studies revealed that bacterial dysbiosis was potentially associated with CRC and could increase CRC occurrence risk [26, 27]. In addition, Lanka et al. pointed out that gut microbiota could be a potential predictor for early postoperative complications and local recurrence [28].

## The direct effect of pathogenic bacteria

The occurrence of CRC is a comprehensive process influenced by genetic factors. It has different pathogenic mechanisms in the following process: 1. The gut microbiota adheres to intestinal epithelial cells. 2. The gut microbiota binds to Toll-like receptors (TLRs), which are located on the membrane of epithelial cells and endosomes [29]. 3. The gut microbiota synthesizes and secretes cytotoxic biomolecules or metabolites [30]. 4. Cytotoxic biomolecules or metabolites can affect DNA structure and repair sufficiency, pericellular stagnation and chromophore malformation [31, 32]. Experimental results have shown that *S.* *bovis* could increase cell proliferation and the secretion of cytokines and transcription factors, such as interleukin-1 (IL-1), interferon-G (IFN-G), IL-8 and nuclear factor kappa-B (NF-κB), which could promote human carcinoma [33]. Gram-negative bacteria could increase the expression of TLR4 [34]. Activated TLR4 may promote CRC occurrence via the Cox-2 or epidermal growth factor receptor (EGFR) signalling pathway and other mechanisms [35]. Li et al. found that the intestinal flora in CRC patients promoted the progression of adenoma, damaged intestinal barrier function, induced chronic low-grade inflammation and activated the Wnt signaling pathway [36].

In healthy people, the intestinal microbiota inhabits the colon mucus without triggering an inflammatory response, which could prevent direct contact with gut microbiota, while Dejea et al. considered that the mucosa-associated microbial community is an important factor in CRC pathogenesis, particularly in the proximal colon [37, 38]. Invasive bacteria can disrupt biofilms and cause a series of carcinogenic effects [39]. A study showed that bacteria were involved in the formation of proximal tumours and not in distal cases, suggesting that CRC could play different roles between the proximal colon and distal colon [40]. The reason could be as follows: first, proximal CRC is more hypermethylated and has a higher mutation rate [41]. Second, the proximal colon has a high level of fermentable carbohydrate substrates, while the distal colon has a thickened mucus layer and increased bacteria, which could attenuate immune activity [42–44]. When the local environment changes, for example, in cases of operation and infection, bacteria are more likely to accumulate in the distal colon [20, 43, 44]. Nevertheless, a recent metagenomic study revealed that distal CRC patients had more abundant carcinoma-associated bacterial genes in the feces than proximal CRC patients [45]. The proximal colon contains more abundant* F. nucleatum*, and their metabolism can induce carcinogens and an inflammatory tumour microenvironment and increase the mutation rate [46]. This phenomenon indicated that site-specific bacteria could cause CRC, and some bacteria play different roles between proximal and distal CRC.

Although *F. nucletum* is an indigenous species in the normal oral cavity, it is associated with pathological changes of the colon and rectum. It has been discovered that higher *F. nucleatum* is associated with higher T stages, lymph node metastases, tumour invasion, and larger tumour sizes [3, 5, 47]. Studies have revealed that *F. nucleatum* is higher in patients with adenomas, high-grade dysplasia and CRC than in healthy people [48]. A higher concentration of *F. nucleatum* has a negative impact on survival outcomes [3, 48–50]. The phenomenon that *F.* *nucleatum* levels were positively correlated with younger age and MSI-H in South Africa was found by Viljoen et al. [51]. According to a large cohort study, activating autophagy to stimulate tumour growth and promote chemotherapy tolerance can lead to a shorter survival time [42]. Furthermore, it is strongly associated with the CpG island methylator phenotype, the density of CD3 + T cells, microsatellite instability and chromosomal instability [51–54]. Gur et al. reported that *F. nucleatum* could inhibit the function of NK cells by binding to the inhibitory receptor TIGIT via the Fap2 protein [55].

ETBF is associated with chronic colorectal diseases, including enteritis, chronic colorectal dysfunction and even CRC [56, 57]. Viljoen et al. revealed that the levels of ETBF and *F. nucleatum* were significantly higher in advanced CRC (stage III and IV) and had a positive association between regional lymph node metastases and high-level colonization by *Fusobacterium* [49, 51]. ETBF triggered β-catenin nuclear signaling, induced c-Myc expression and cellular proliferation and increased colitis and tumors in a Min/+ mouse model [58, 59]. ETBF promotes CRC in the following ways. First, NF-κB plays an important role in the host response to microbial infection by coordinating innate and acquired immune functions [60]. ETBF can stimulate intracellular IL-17 secretion, which activates the NF-κB protein, triggering long-term chronic inflammation and finally leading to tumorigenesis [61]. Furthermore, the activated NF-κB signalling pathway could increase chemokine (CXCL1, CXCL2, and CXCL5) and polyamine metabolism and induce DNA damage [62]. Second, the mitogen-activated protein kinase (MAPK) signaling pathway regulates cell inflammation, proliferation and apoptosis [63]. Under bacterial stimulation, intestinal epithelial cells rapidly secrete IL-8 and monocyte chemotactic protein 1 (MCP-1), which activate the MAPK signaling pathway. This pathway induces intercellular adhesion molecule-1 (ICAM-1) and the enhancement of adhesion between inflammatory cells and endothelial cells, which could decrease the permeability of inflammatory cells, aggravate the inflammatory response, inhibit cell apoptosis and further promote the development of CRC [64]. Third, ETBF also increases the expression of cyclooxygenase (COX)-2 and releases prostaglandin E-2 (PGE2), which activate inflammation associated with signal transducers and activators of transcription (STAT)3 and interact with epithelial cells [65]. Toxin can degrade E-cadherin, thus altering signalling pathways, upregulating spermine oxidase and leading to cell morphology, promoting carcinogenesis and irreversible DNA damage [65]. Finally, ETBF can also promote tumor growth via associated lncRNA1 by activating Ras homologs [66].

*S.* *bovis*, also named *Streptococcus* *gallolyticus* subsp. *gallolyticus* (SGG) is one of the first bacteria clearly associated with CRC occurrence [67]. It is more common in early-stage adenomas than in later-stage carcinomas, and its concomitant inflammatory factors concentrate in the intestine by recruiting CD11b^+^TLR-4^+^cells [68, 69]. Meamwhile, Aymeric et al. concluded that CRC-specific conditions promote *S.* *bovis* colonization by the Wnt pathway and the decreased expression of the bile acid apical transporter gene Slc10A2 [67]. *Bovis* has also been widely studied, and its antibody may serve as a potential marker for CRC at an early stage of disease [70].

## Inflammatory factor

Numerous inflammatory factor were found to be associated with protumor or antitumor effects in CRC pathogenesis [71, 72]. IL-1 promotes tumorigenesis and tumor metastasis in CRC [73], and the tumor-suppressive effects of IL-1 have also been reported [73, 74]. Tumor necrosis factor (TNF) plays a dual role in cancer. It can induce apoptosis by activating the TNF receptor and promote tumorigenesis and cancer progression by activating TNF receptor 2 [71]. Gut microbiota dysfunction could induce abnormal immune responses and create a special immune microenvironment causing DNA damage and gene mutation. For example, members of *Enterobacteriaceae* are known to cause inflammation in the gastrointestinal tract and contribute to CRC [75]. IL-8 has a chemotactic effect on neutrophils, recruiting more immature polymorphic cells and further aggravating inflammation and cell damage [76]. Tseng et al. showed that the IL-6 signaling pathway plays an important role in the occurrence and chemoresistance of various cancers, including CRC [73, 77, 78]. Under inflammatory conditions, NF-κB-induced IL-6 can lead to cancer progression and metastasis via the IL-6/STAT3 signaling pathway [77]. Bacteria-derived lipopolysaccharide stimulated IL-1β- and IL-17-producing T helper cell activation to promote inflammation [79]. By using human and mouse models, Sobhani et al. demonstrated that CRC-associated microbiota induced gene methylation. Several gene promoters were hypermethylated in CRC but not in normal tissues [27]. Hypermethylation of the promoter Wif1 could serve as a surrogate diagnostic marker for early CRC [80].

In contrast, some bacterias could enhance the secretion of anti-inflammatory factors, such as IL-10 and TGF-β, which reduce the concentrations of proinflammatory factors, such as IFN-γ, reduce the infiltration of NK cells and the degree of the inflammatory response [81, 82]. By focusing on TGF-β1 signaling, IL-25 is constitutively produced by gut mucosal cells and restrain intestinal inflammation [79], and IL-6R is considered a potential target for CRC treatment. Tocilizumab, an IL-6R antagonist antibody, blocks the IL-6/STAT3 signaling pathway, which could reduce CRC viability and enhance cell apoptosis [83].

## Effect of bacterial metabolites

The gut microbiota participates in synthesis and metabolism, while the metabolites regulate intestinal micro ecological balance in return. For example, red meat, processed meat and high protein food are digested by enzymes into toxic nitrogen and sulfur-containing substances [84]. Hydrogen sulfide (H2S), generated by sulfate-reducing bacteria (SRB), can promote inflammation of the colon mucosa, induce DNA damage and methylation, release free radicals and simultaneously inhibit the synthesis of cytochrome oxidase butyric acid and mucus [84, 85]. In addition, a high-fat diet can produce secondary cholic acid, affecting mitosis, activating NF-κB and epidermal growth factor receptor (EGFR) and promoting tumour development [69, 86, 87]. Intestinal compounds activated by microorganisms are derived from some vegetable foods, such as intestinal lignans, which may play a role in carcinogenesis [88]. For example, intestinal microbes can produce short-chain fatty acids (SCFAs), such as butyric acid, by fermenting food, which has been proven to inhibit intestinal inflammation, regulate the immune response, maintain barrier function, reduce precancerous lesions and regulate DNA methylation [27, 89]. SCFAs generated by intestinal microorganisms are primary nutritional substrates for colonocytes [90]. By upregulating secretory IgA and cytokines, prebiotics can enhance host immunity [91]. Butyric acid protects against pathogenetic mechanisms mediated by reactive oxygen species and aids in understanding the apparent protection against CRC. Butyrate is important for colon epithelial cells by increasing apoptosis, decreasing cell proliferation and increasing differentiation [92]. Butyrate substrates have been shown to increase the acute apoptotic response and halt the cell cycle through animal studies [93]. Acting as a histone deacetylase inhibitor, butyrate could also regulate the expression of CDKN1A [94]. They can also increase phytochemicals from active microorganisms, such as polyphenols with anti-inflammatory and antioxidant properties [16]. Several species, such as *Lactobacillus* *rhamnosus* GG, *Lactobacillus*, *Lactobacillus* *casei*, and *Bifidobacterium* *breve*, can also downregulate inflammatory reactions or preserve intestinal barrier function [95, 96]. It has also been reported that the protective protein p40 can inhibit epithelial cell apoptosis and intestinal barrier destruction induced by cytokines and enhance IgA secretion [97]. Lactate dehydrogenase or other molecules from lactic acid bacteria can induce apoptosis or inhibit CRC invasion [87, 98]. However, some reports hold the opposite view: butyrate treatment could promote epithelial cell proliferation and polyp formation and eventually tumor progression [99]. Therefore, butyric acid supplementation does not necessarily benefit the host’s health, which is highly dependent on the somatic genetic background [100].

## Effect of antibiotics

Antibiotics might be related to inhibiting tumour proliferation, invasion and growth. For patients with colon cancer, antibiotic therapy is even recommended as an immunotherapy strategy [100, 101]. After antibiotic manipulation of the gut microbiota, a reduction in tumor load was detected, which supported the hypothesis of bacterial involvement in carcinogenesis [102]. For example, metronidazole can eradicate *Fusobacterium* colonization and inhibit the proliferation of CRC [103]. In the future, antibiotic therapy might become a potentially valuable method to prevent cancer. However, accumulating evidence indicates that antibiotics can damage immunotherapy and induce disease progression by further creating microbial disorders. A meta-analysis including 8 studies showed that current research only suggests a weak association between cumulative antibiotic consumption and risk of CRC because of the heterogeneity and quality of the available research [104, 105]. In addition, antibiotic-mediated bacterial clearance may aggravate unexpected adverse reactions; for example, vancomycin and anti-ctla-4 can lead to more severe and fatal colonic inflammation in mice with colitis [106]. Therefore, a large number of high-quality studies are needed to further confirm the relationship between antibiotic exposure and CRC progression.

## Treatment of CRC by regulating gut microbiota

The theory of the “driver-passenger” model supposes that disease-related microorganisms may cause DNA damage, increase cell proliferation, produce genotoxic substances and lead to adenoma or cancer [75, 107]. The microenvironment of the tumour changes, which may be beneficial to the growth of bacteria that dominate the tumour. In recent years, an increasing number of people have paid attention to the intestinal flora in different stages of CRC to distinguish the initial flora from the late flora [75, 108]. Thousands of bacteria lie in the intestinal tract, but not all of them are carcinogens. Probiotics are active intestinal microorganisms that bring health benefits to hosts [109]. In addition to direct interaction, probiotics also produce metabolites, such as acetic acid or bacteriocin, which can inhibit the growth of pathogens by lowering the pH value and play a direct antibacterial role [110–112].

The most widely used clinically adopted probiotics include *Lactobacillus*, *Bifidobacterium* and *Clostridium* *butyricum*. Zaharuddin et al. showed that probiotics may modify the intestinal microenvironment, resulting in a decline in proinflammatory cytokines [113]. Geier et al. discovered that lactic acid bacteria and *Bifidobacterium* could decrease CRC incidence by reducing the activities of azo reductase, nitro reductase and β-glucuronidase [109]. The expression of Th17 cells and the secretion of IL-23 and IL-17 were downregulated by lactic acid bacteria by inhibiting the STAT3 and NF-κB signalling pathways [114]. Another study showed that *Lactobacillus rhamnosus*, *Lactobacillus plantarum* and *Escherichia coli* enhanced intestinal barrier function by upregulating the expression of tight junction proteins, stimulating mucin production and promoting epithelial restitution [115–117]. Lactic acid bacteria showed antitumour properties in a CRC mouse model induced by dimethylhydrazine (DM*H)* [105]. By affecting the metabolism of retinoic acid, *Bifidobacterium infantis* and *Bifidobacterium breve* activate intestinal dendritic cells (DC) and release regulatory T cells (Treg) expressing Foxp3 + , regulatory T cells of type 1 (Tr1) and il-10, thus exerting an antitumour effect [115, 116]. Oral probiotics (such as *Bifidobacterium* and *Akkermansia* *muciniphila*) and fecal microbial transplantation (FMT) can significantly enhance immune therapy based on PD-1 by enhancing the response of dendritic cells and T cells [118–120]. In animal experiments, Sivan et al. found that oral *Bifidobacterium* in mice had the same efficacy as an anti-PDL1 immunosuppressant and almost completely inhibited the growth of melanoma in mice [120].

The anti-inflammatory effect can also be produced by gene modification and protein expression. For example, efforts are devoted to studying S-layer proteins, lipoteichoic acid and exopolysaccharides, and the deletion of lipoteichoic acid and immunostimulating protein in lactic acid bacteria downregulates the expression of proinflammatory mediators and inhibits the occurrence of colonic inflammation and cancer [110, 121]. Intestinal microbes recovered through whole metagenomic analysis have become a potential strategy for the prevention and treatment of CRC [27, 105]

## FMT

The gut microbiota creates a complex microenvironment that can influence the tumor in a very heterogeneous way that relies on intrinsic heterogeneity [77]. Studies based on the intestinal flora of healthy people and patients show that FMTs can restore microbial homeostasis and may help to improve various gastrointestinal diseases, including irritable bowel disease and *Clostridium* *difficile* infection [122]. FMT seems to bring some benefits. For example, it increases the diversity of microorganisms without destroying the intestinal flora. FMT of A. shahii had a good response in antibiotic-treated colon tumor mice [123]. In addition, FMT led to a reduction in the number of tumors and inflammation, as well as the inhibition of proinflammatory molecules (IL-1β, IL-6, and TNF-α) and an increase in anti-inflammatory cytokines (IL-10 and TGF-β) [79, 124].

However, from colon cancer mice or CRC patients to sterile mice, FMT experiments revealed that intestinal microflora played a key role in the development of CRC [125, 126]. This treatment has the risk of transferring pathogens and antibiotic-resistant genes, and some people worry that the donor’s microflora and its hidden complexity may cause chronic diseases in Shinjuku [127]. Nooij et al. studied the change in the prevalence and abundance of potentially carcinogenic pks + *E.* *coli* after FMT [128]. The pks genome island encodes colibactin, which is a polypeptide-polyketone hybrid that can induce double-stranded DNA breakage and chromosome aberration [129]. At present, there is no validated diagnostic test that can accurately assess the carcinogenic potential of microorganisms. Importantly, all medical decisions require systematic risk and benefit assessment [127]. FDA warned of the potential risks of spreading multidrug-resistant bacteria and subsequently developing life-threatening infections. In a case reported by the FDA, two immunocompromised patients were infected by broad-spectrum β-lactamase (ESBL)-producing *E. coli*, and one patient died [130]. The evidence of using FMT in cancer is limited, and more clinical trials of solid cancer are just beginning to appear. The risk of adverse events should not be underestimated, including potentially fatal systemic inflammatory response syndrome and unintentional transfer of pathogens, including highly resistant microorganisms [123].

In general, the continuous changes after FMT show that the long-term consequences, whether promising or harmful, should be taken into account [131]. Rooks and others emphasized the importance of FMT in preventing, treating and controlling disease progression by changing the intestinal flora [132]. If FMT could be used to predict CRC and adopted in clinical practice, it could be a novel, convenient, efficient, economical and noninvasive method.

## Conclusion

At present, the microorganisms promoting CRC mainly include nuclear *F.* *nucleatum*, ETBF, and *S.* *bovis*, and the antitumour microorganisms mainly include *Lactobacillus* and *Bifidobacterium*. The increased content of self auto-induced factor 2, extracted from *F.* *nucleatum* in CRC patient feces, was associated with tumor immunity through tumor-associated macrophages and the CD4/CD8 ratio. This indicates that it may be a potential marker for clinical screening [133]. Another study pointed out that combining IgA and IgG against *Fusobacteria* with CEA and CA 19–9 may be a better method for screening CRC. (AUC = 0.743, specificity = 94.22%, sensitivity = 40.00%) [134]. It is challenging to select a single intestinal microorganism as the diagnostic marker, and the combination of multiple microorganism scans, such as *Roseburia*, *Clostridium* and *Akkermansia*, improves the sensitivity and specificity [36]. In the future, it is possible to formulate a new standard for early screening and recurrence by the flora mentioned above [122, 135].
